# Quantitative Microbial Risk Assessment for Workers Exposed to Bioaerosol in Wastewater Treatment Plants Aimed at the Choice and Setup of Safety Measures

**DOI:** 10.3390/ijerph15071490

**Published:** 2018-07-14

**Authors:** Annalaura Carducci, Gabriele Donzelli, Lorenzo Cioni, Ileana Federigi, Roberto Lombardi, Marco Verani

**Affiliations:** 1 Laboratory of Hygiene and Environmental Virology, Department of Biology, University of Pisa, Via S. Zeno 35/39, 56127 Pisa, Italy; annalaura.carducci@unipi.it (A.C.); gabriele.donzelli@for.unipi.it (G.D.); marco.verani@unipi.it (M.V.); 2 Scuola Normale Superiore of Pisa, Piazza dei Cavalieri 7, 56126 Pisa, Italy; lorenzo.cioni@sns.it; 3 Department of Technology Innovations, Research, and Certification Sector—INAIL, Research Centre—Via Fontana Candida 1, 00040 Monte Porzio Catone (Rm), Italy; ro.lombardi@inail.it

**Keywords:** human adenovirus, quantitative microbial risk assessment, wastewater treatment plants

## Abstract

Biological risk assessment in occupational settings currently is based on either qualitative or semiquantitative analysis. In this study, a quantitative microbial risk assessment (QMRA) has been applied to estimate the human adenovirus (HAdV) health risk due to bioaerosol exposure in a wastewater treatment plant (WWTP). A stochastic QMRA model was developed considering HAdV as the index pathogen, using its concentrations in different areas and published dose–response relationship for inhalation. A sensitivity analysis was employed to examine the impact of input parameters on health risk. The QMRA estimated a higher average risk in sewage influent and biological oxidation tanks (15.64% and 12.73% for an exposure of 3 min). Sensitivity analysis indicated HAdV concentration as a predominant factor in the estimated risk. QMRA results were used to calculate the exposure limits considering four different risk levels (one illness case per 100, 1.000, 10.000, and 100.000 workers): for 3 min exposures, we obtained 565, 170, 54, and 6 GC/m^3^ of HAdV. We also calculated the maximum time of exposure for each level for different areas. Our findings can be useful to better define the effectiveness of control measures, which would thus reduce the virus concentration or the exposure time.

## 1. Introduction

Occupational exposure to bioaerosols in workplaces can produce a wide range of health effects, such as infectious diseases, acute toxic effects, inflammatory, and allergic diseases, chronic obstructive pulmonary diseases (COPD), fetal harm, and cancer [1,2]. Bioaerosols are biological particulates suspended in air that contain a large amount of fungi and bacteria as intact microbes or fragments such as endotoxins [3,4] as well as viruses [5]. Studies have revealed the association between exposure to bioaerosols and the onset of respiratory health outcomes during waste management activities [6]. 

The European Union (EU) Directive 2000/54/EC [7] on the protection of workers establishes that, in the case of any activity likely to involve an exposure to biological agents, the risk must be assessed and the measures to control it must be defined. Then, every person, even if only potentially exposed to pathogens, must be protected using the best practices based on the most up-to-date scientific knowledge and on the current level of technological development.

The EU Directive 2000/54/EC does not define the type of approach that has to be used. Presently, risk assessment is normally carried out by establishing the risk levels based on the probability and magnitude of the consequences deriving from exposure and designing a risk matrix where such levels are represented [8] in order to choose and prioritize preventive actions. However, biological risk management is hampered by the lack of efficient quantitative methods for an accurate assessment of the risk factors [9]. Moreover, infection risk levels are commonly estimated by the measurement of bacterial indicators, not pathogens directly linked to illnesses. Quantitative microbial risk assessment (QMRA) is used to evaluate microbiological hazards based on the measure of index pathogens in environmental matrices and their dose–response relationships. This is a well-established decision support tool in the fields of water supply, water reuse, and water recreational [10] and food safety [11,12], but it could also be applied to risk analysis in workplaces, in particular, for exposure to bioaerosols, although at present, it is not currently used. A tentative approach of this application was recently developed regarding exposure to the human adenovirus (HAdV) through bioaerosol in different settings [13]. In that case, the model used point estimate values of HAdV concentrations to assess the risk without taking into account uncertainty and variability. We choose HAdV as the reference pathogen because it is extremely widespread in wastewaters, it is known to be very resistant to water treatment, and it is likely to remain infectious in the environment for a long time. Moreover, it can cause both respiratory and gastrointestinal infections and it can be transmitted through ingestion as well as inhalation, followed or not by the swallowing of mucous [14]. 

In the present work, in order to perform a reliable occupational biological risk assessment, we developed a probabilistic QMRA model for the exposure of workers to bioaerosols containing HAdV using a dose–response model recently developed to describe the distributions of infectivity and pathogenicity in various challenge studies incorporating differences in inoculation routes [15,16]. 

The considered work settings were wastewater treatment plants (WWTP) that produce highly contaminated aerosol as demonstrated by numerous studies [17,18,19]. Specifically, the final aim was to obtain useful information for setting up practical risk management interventions and strategies: to compare the risk of infection and illness in different areas of the plant and for different exposure times; to provide evidence of the most important variables influencing the risk; and to try to establish critical values for different risk management options.

## 2. Materials and Methods

QMRA was conducted following a static approach [20], assessing health risk at the individual level. Thus, secondary transmission of infection/disease and immunity to infection from microbial agents were not taken into account. The QMRA framework includes four steps [20], which are briefly described below.

### 2.1. Hazard Identification and Exposure Assessment

The objective of the exposure assessment model was to estimate the dose of HAdV to which workers are exposed while performing their working activities. The data on HAdV genomic copy concentrations were obtained from a study [13] that measured bioaerosol contamination in different areas of WWTPs. Briefly, air samples (up to 3000 L) were collected with an impactor sampler loaded with Rodac plates containing tryptone soy agar that were subsequently eluted in 3% beef extract. The eluate was centrifugated and the supernatant was decontaminated. Finally, the viral DNA was extracted and HAdV was detected with quantitative PCR [13].

The Easy-Fit statistical package (MathWave Technologies, version 5.6) was used to obtain the probability distributions of the concentration data.

The HAdV dose that each worker is exposed to by inhalation during a definite exposure time was estimated from Equation (1) using input parameters, each with its own probability distribution, drawn from the literature—except the exposure time, which was the independent variable in the simulations:

(1) $$dose=C_{HAdV}/r_{eff}\times t_{exp}\times f_{conv}\times r_{in}\times cf$$

In Relation (1): *dose* is the amount of HAdV inhaled (infectious viral particles/person/single exposition); *C_HAdV_* is the concentration of the genome copies (GC) of HAdV detected in air samples (GC/m^3^); *r_eff_* (recovery efficiency) is the recoverable amount of HAdV acutally present in the sample, characterized by a triangular distribution with parameters 0.19, 0.403, 0.68 [21]; *t_exp_* (exposure time) is the amount of time that the worker is exposed to bioaerosol (minutes), characterized by a uniform distribution on the range interval (from 0 to 100); *f_conv_* (conversion factor) is the ratio between tissue culture infective dose (TCID_50_) and GCs measured in TCID_50_/GC, with 1 TCID_50_ = 700 GC [22]; *r_in_* (inhalation rate) is the volume of air inhaled per unit of time (m^3^/hour), characterized by a log-normal distribution with a mean value of 1.40 and a standard deviation of 0.51 [23]; and cf is a constant conversion factor equal to hour/60 min.

### 2.2. Dose–Response Assessment and Risk Characterization

We adopted the dose–response model recently developed by Teunis et al. [15] that combines dose–response data from human volunteer studies concerning three adenovirus types (AdV4, AdV7, AdV16) inoculated by four different pathways (oral ingestion, inhalation, intranasal, and intraocular droplet inoculation). In our work, we used dose–response parameters (α, β, η, r) that refer to the inhalation route. This is a QMRA approach that is not based on the direct measurement of illness cases (as for epidemiological studies) but on the measurement of pathogen dose with health effects that are inferred using known mathematical dose–response relationships.

Probability of infection (*P_inf_*) is represented by Equation (2) where _1_F_1_ denotes a confluent hypergeometric function of the first kind and where the heterogeneity in the host–pathogen interaction is considered. Equation (3) defines the dose–response relationship for the probability of acute symptoms in infected individuals or the conditional probability of illness, given infection (*P_ill/inf_*), that is, pathogenicity. Then, the unconditional probability of illness as a function of dose (*P_ill_*) is given [15]:

$$P_{ill}=P_{inf}\times P_{ill|inf}.$$

where, for the probability of infection, we have the following relation:

(2) $$P_{inf}=1-{}_{1}\;F_{1}\;\left(\alpha ,\;\alpha +\beta ;-dose\right)$$

In such a relation, we have the infectivity parameters for the adenovirus dose–response α = 5.24 and β = 2.95 measured in TCID_50_. On the other hand, for the conditional probability of illness, given infection, we have the following relation:

(3) $$P_{ill|inf}=1-{\left(1+\frac{dose}{\eta }\right)}^{-r}$$

In Relation (3), we have the pathogenicity parameters for the adenovirus dose–response η = 3.36 measured in TCID_50_ and r = 3.04 dimensionless.

### 2.3. Model Implementation

We implemented the models with the Vensim^®^ simulation software [24]. In the models, we set up Relations (1), (2), and (3) together with all the other relevant mathematical relations, for instance, those for the simulation of the concentrations of HAdV in the various areas and the implementation of the hypergeometric functions. On the various models, we performed a simulation for each distribution and for its average value for 6000 iterations. We selected such a value since we assumed it was high enough to allow the appreciation of the trends of the various probability functions (2) and (3). Afterward, a simple univariate sensitivity analysis [25] was performed on three inputs parameters—inhalation rate, recovery efficiency, and concentration of HAdV—in order to understand the importance of each of them on the model output (*P_ill_*). The value of each parameter was varied, one at a time, along with the uncertainty range of that parameter in order to determine the effect on the final risk estimate. Moreover, the QMRA model was used in order to estimate, for each exposure scenario, the thresholds exposure times for each area to reach four different levels of probability of illness *P_ill_* = 10^−5^, 10^−4^, 10^−3^, 10^−2^. The exposure scenarios were different areas of the WWTP, characterized by different probability distributions of the concentration of HAdV or its average value, that were used in the dose calculation (see Relation (1)). Finally, the values of the concentrations needed to attain the same levels of probability of illness after 3 min of exposure were estimated. These results can be useful to select the best prevention measures for workers: reduction of exposure time, reduction of environmental contamination, or use of respiratory protective equipment (RPE).

All figures were generated with Excel for Windows (Microsoft Office Excel 2016, Redmond, Washington, DC, USA).

## 3. Results

In the considered WWTP, we found four areas, each one characterized by a different contamination level [17]. For each area, we obtained the following best-fitting distribution type of the concentration of HAdV (measured in GC/m^3^ and with μ denoting the average value and σ the standard deviation):

1. For the *sewage influent*, a lognormal distribution with µ = 7.7968 and σ = 1.5946;
2. For the *biological oxidation tank*, a lognormal distribution with µ = 7.4005 and σ = 1.7299;
3. For the *sludge treatment*, a uniform distribution in the range [306.67, 1664.9];
4. For the *side-entrance manhole*, a uniform distribution in the range [306.67, 1664.9]. With the side-entrance manhole, we refer to the area of access to the manholes for inspection operations.

We used these distributions as well as their average values in the models for the calculation of the dose (see Relation (1)).

**Figure 1:**
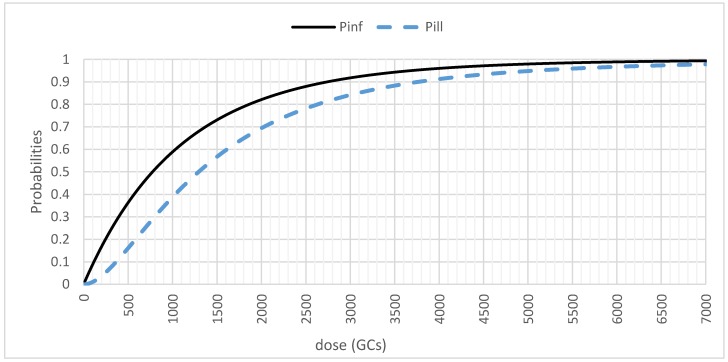
Infection (*P_inf_*) and illness (*P_ill_*) probabilities (on a linear scale) as a function of the dose in genomic copies (GCs).

In Figure 1, we present the relationships between the dose (in GCs) and the probabilities of infection (*P_inf_*) and illness (*P_ill_*). We made the dose vary linearly from a minimum to a maximum value, calculated from previous simulations with real parameters. All of the other parameters were kept to their average values.

If we consider an exposure time of 100 min, in the four areas different doses were reached:

1. 60.45 TCID_50_ or 42,315 GC at the biological oxidation tank;
2. 72 TCID_50_ or 50,400 GC at the sewage entrance;
3. 8.15 TCID_50_ or 5705 GC at the sludge treatment;
4. 5.8 TCID_50_ or 4060 GC at the side entrance manhole.

**Figure 2:**
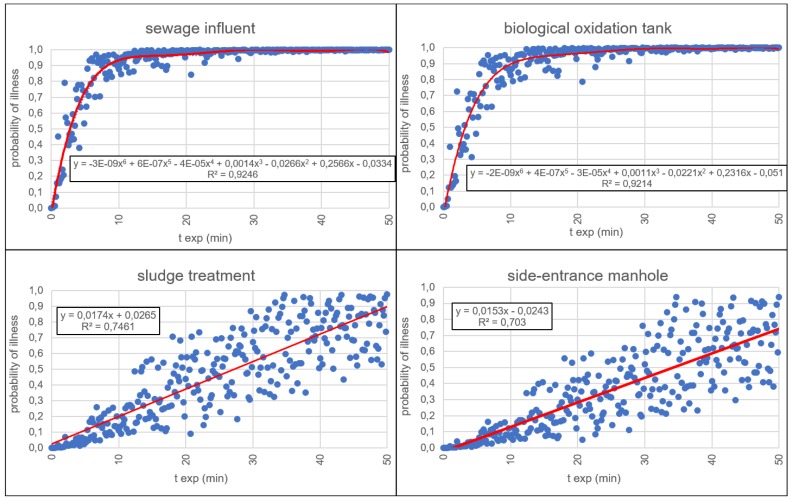
The probability of illness *P_ill_* as a function of the exposure time (*t_exp_*) for each exposure event in the different areas over an interval of time long enough (50 min) in order to appreciate the trend lines. In the equations, y corresponds to the probability of illness and x to *t_exp_*.

Figure 2 shows the relationships between the probability of illness (*P_ill_*) and the exposure time (*t_exp_*) for the four different areas of the WWTP. In environments with higher values of the concentration (i.e., sewage influent and biological oxidation tank), the probability of illness follows a six-grade polynomial trend line with a very good reliability (*R*^2^ = 0.92). Otherwise, with lower values of the concentration, the probability of illness follows an almost linear trend line (with *R*^2^ = 0.74 for sludge treatment and *R*^2^ = 0.70 for side entrance manhole).

**Figure 3:**
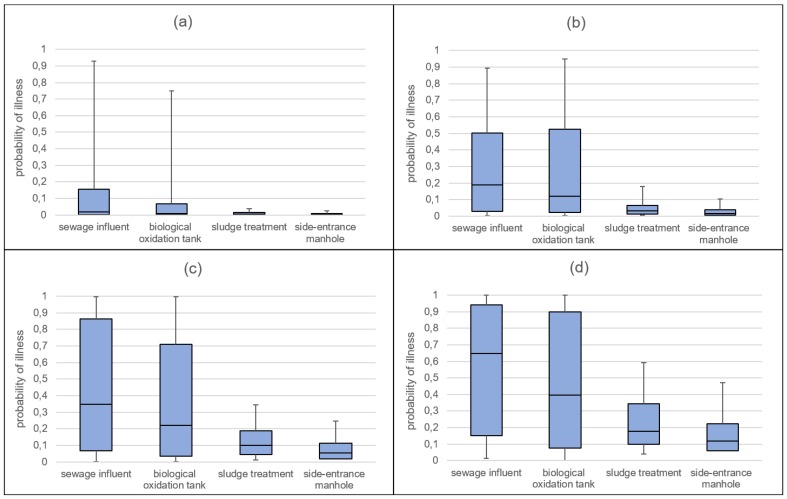
Probability of illness *P_ill_* (5th, 25th, 50th, 75th and 95th) in the considered work settings and for different exposure times: 0–3 min (**a**); 3–5 min (**b**); 5–10 min (**c**); 10–15 min (**d**).

In Figure 3, the probability of illness *P_ill_* is represented according to different values of the parameter *t_exp_* in the four WWTP areas. For an interval of time from 0 to 3 min, the average risk values were equal to 14.64%, 12.73%, 1.13%, and 0.7%, respectively, for sewage influent, biological oxidation tank, sludge treatment, and side entrance manhole areas. For the next time intervals (from 3 to 5, from 5 to 10, and from 10 to 15 min), the average risk values increase, as well as their variability in terms of interquartile range, much more for the most contaminated areas.

### 3.1. Sensitivity Analysis 

**Figure 4:**
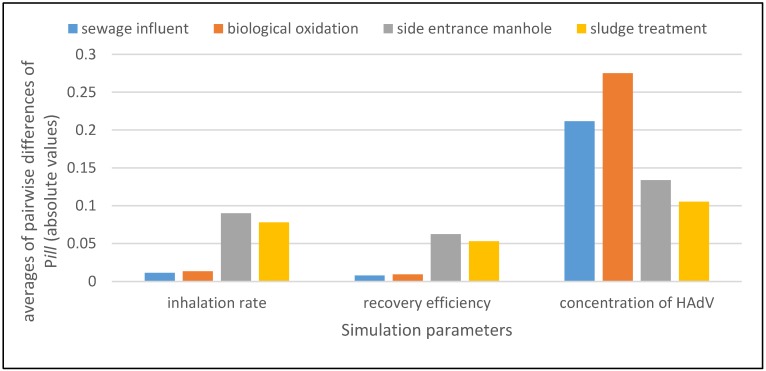
Sensitivity analysis determines how a change in each input parameter (inhalation rate *r_in_*, recovery efficiency *r_eff_*, and concentration of HAdV *C_HAdV_*) affects the change of the probability of illness.

Figure 4 shows, for each parameter (on the x axis) in each occupational environment (in the legend above the histogram), the averages (on the y axis) of the absolute values of the pairwise differences of the values of the model output *P_ill_* between the case where all the parameters (i.e., inhalation rate, recovery efficiency, and concentration of HAdV) are held at a constant value, and the case where only one of them is varied at a time according to its assumed probability distribution (through which we model either an uncertainty or a variability). The used probability distributions are as follows: log-normal distribution for inhalation rate, triangular distribution for recovery efficiency (see after Relation (1)), and HAdV distribution proper for each working setting.

It is evident how the parameter *P_ill_* is most sensitive to variations of the parameter *C_HAdV_*.

### 3.2. Application of Quantitative Microbial Risk Assessment to Support Risk Management

**Figure 5:**
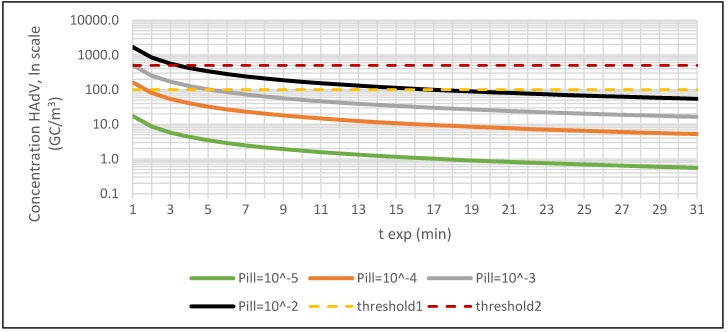
Iso-probability illness curves obtained for different combinations of exposure time and concentration of HAdV (threshold1 and threshold2 are examples of HAdV concentrations).

QMRA models were used to try to estimate the exposure thresholds, that are the upper limits of *C_HAdV_* for the areas of WWTPs, corresponding to four probabilities of illness (*P_ill_* = 10^−2^, 10^−3^, 10^−4^ and 10^−5^). The same risk levels, represented in Figure 5 by four iso-probability curves, can be obtained from a different combination of exposure time (*t_exp_*) and *C_HAdV_* according to the following relation:

*t_exp_* × *C_HAdV_* = *k*(*P_ill_*) (4)

where *k*(*P_ill_*) is a constant that takes higher values the higher the value is of *P_ill_*. 

We defined a dose value for each *P_ill_* threshold (10^−2^, 10^−3^, 10^−4^, 10^−5^), based on a simulation for sewage influent, chosen as an example. Then, *k*(*P_ill_*) was rewritten as relation (1) as follows:

*k*(*P_ill_*) = dose × *r_eff_*/(*f_conv_* × *r_in_* × *cf*). (5)

Finally, *C_HAdV_* was calculated from Equation (4) using *k*(*P_ill_*) from Equation (5) and varying *t_exp_* from 1 to 31 min.

It is clear that in every iso-probability curve, for a fixed value of the *P_ill_*, the *C_HAdV_* decreased gradually with the increase of the exposure time *t_exp_* and vice versa.

Figure 5 represents the exposure curve for a general scenario. In Figure 5, we show also two examples of threshold values (threshold1 = 100 GC/m^3^ and threshold2 = 500 GC/m^3^) that can be used in order to identify a critical value of the exposure time for each defined level of risk as a predefined value of *P_ill_*. For threshold1, we have about 2 min for a level of risk of 10^−4^, about 5 min for a level of risk of 10^−3^, and about 17 min for a level of risk of 10^−2^. For threshold2, we have about 1 min and 4 min, respectively, for the levels of risk of 10^−3^ and 10^−2^.

Through the use of QMRA models, it has been possible to determine the maximum values of the exposure time *t_exp_* during which the workers can stay in an area with a *C_HAdV_* of 100 GC/m^3^ without using RPE to maintain the risk below the different threshold values of *P_ill_* (10^−2^, 10^−3^, 10^−4^, and 10^−5^). For the sewage influent, the theoretical exposure times would be equal to 12 s, 4 s, 1.5 s, and 0 s, respectively. For the biological oxidation tank, the exposure times would be equal to 14 s, 4 s, 1.4 s, and 0 s, respectively. Then, in these first areas, the workers should always wear an RPE. For the sludge treatment, the exposure times would be 1.72 min, 31 s, 10 s, and 3 s, respectively, and for the side-entrance manhole, the exposure times would be 2.25 min, 44 s, 14 s, and 4 s. Again, also in these areas, the workers should always wear an RPE.

Analogously, for a fixed exposure time, it is possible to estimate from Figure 5 the upper limits for the parameter *C_HAdV_*: For example, for 3 min of exposure in any working area, the thresholds in *C_HAdV_* could be estimated at 6, 54, 170, and 565 GC/m^3^, respectively, for *P_ill_* = 10^−5^, 10^−4^, 10^−3^, and 10^−2^.

## 4. Discussion

In the present study, QMRA analyses showed a high-risk of illness for wastewater workers posed by exposure to bioaerosol, confirming the findings of the existing epidemiological studies about the association between employment in sewage treatment plants and work-related adverse health effects [26,27]. As expected, the risk was higher for the most contaminated areas and increased with the exposure time, but the advantage of using the model was the possibility of calculating more precisely the risk values corresponding to different combinations of contamination and times and comparing them with definite risk levels. Therefore, the application of QMRA provides a quantitative basis for the decision-making process in occupational risk management. Concretely, the variables that can be controlled from a risk management perspective are the exposure time and the concentration of biologic agents in the different areas of the WWTPs. QMRA models could be used to determine the times of exposure corresponding to various levels of risk and then to identify when the use of specific RPE becomes necessary. In addition, the identification of high-risk areas for workers could be useful to establish when to reduce bioaerosol emissions and dispersion, with interventions such as ventilation [28], disinfection treatments, or the coverage or change of the aeration systems used in the biological treatment. The models could also be used to select the most suitable RPE considering its efficiency of protection [29]. 

At present, QMRA is rarely applied to occupational airborne exposure and mainly limited to farmers involved in land application of biosolids or manures [30,31,32]. In the present work, QMRA is included in occupational risk management in order to prioritize and define the control measures needed to reduce the risks below established limits. To the best of our knowledge, this approach has never been applied before to workplaces, but it could be very useful for occupational hygiene, where biological risk assessment is usually carried out in a qualitative or semiquantitative way [33]. 

Another aim of this study was the formulation and the setting of a general model for risk characterization that could be implemented in different working settings. This approach could represent an innovation in the context of occupational risk management for biological hazards because precise risk assessment allows for the design and prioritization of interventions and control measures. In this way, it would be possible to define accurately the biologic risk factors in order to perform the proper evaluation and the subsequent management of risk through the tuning of both individual and collective safety measures (through an accurate choice and use, for instance, of individual protection devices for a given type of risk). Therefore, QMRA provides a risk analyst with quantitative information to better manage worker safety in various occupational settings as a complement to the existing guidance and laws, taking into account technological innovations and the current scientific knowledge.

At present, there are no occupational exposure limits (OELs) for biological agents [34], which impedes decision making for the implementation of strategies for biological risk management in workplaces. The use of QMRA models could be extremely useful to aid the determination of the OELs based on an acceptable level of risk. Nevertheless, the current EU directive [7] does not consider this possibility because an acceptable level of risk is not defined and the risk assessment is based only on the “potential” exposure and on the adopted preventive measures. 

Although the QMRA approach would be extremely promising in work settings, it is affected by some limitations and requires further study. Firstly, the identification of the index pathogen to be used remains a crucial point. In fact, bioaerosol contains a high number of different microorganisms [35] and it is not feasible to apply QMRA models to each of them. In our study, we used HAdV, but the same methodology could be applied to other microorganisms, depending on their epidemiological importance and provided that dose–response curves are known. Moreover, a multipathogen risk assessment could be performed, as is already done in other settings (i.e., recreational waters [36,37,38]). 

Secondly, the values of pathogen concentrations are difficult to obtain in routine monitoring, in which, generally, total bacterial count (TBC) or fecal indicator bacterial (FIB) are measured. Then, it would be very practical to use these parameters in order to estimate the pathogen concentrations, but this would imply the knowledge of relations between them, which is obtainable only with specific monitoring studies. The adoption of a pathogen:indicator ratio introduces a further element of uncertainty in the QMRA model inputs, but it has been reported in the current WHO guidelines for QMRA [39] and has been employed by many authors for the estimation of health risks from exposure to recreational waters [40,41] or wastewater reuse [42,43].

Even if pathogens are directly detected, the most used analytical methods to this aim are the biomolecular ones that generally do not allow for the estimation of infectivity. Then, the relations between genomic copies and infective particles should be determined with specific studies. 

Moreover, occupational exposure via inhalation to airborne microorganisms is responsible for both respiratory and enteric symptoms, owing to swallowing of mucous. To take into account gastrointestinal outcomes, the QMRA model should differentiate the HAdV dose through different exposure routes [44], using specific model parameters for ingestion and inhalation. These additional enteric illness cases should be considered in future research developments.

Finally, other features should be considered for more precise risk assessment, such as the differences among workers based on gender, age, health condition, and immunity. 

Moreover, in our model, deposition efficiency of aerosols in the respiratory tract was not included in the calculation of pathogen dose, assuming that all airborne viruses in aerosol droplets are small enough to be deposited on the bronchial and alveolar region. This aspect should be considered, as reported by Lim et al. [45], in a QMRA study on harvested urban stormwaters for domestic applications.

In fact, our results showed an important level of risk for illness compared to epidemiological studies because our QMRA model explored the health risk of sewage workers at the individual level without considering immunity, which could be responsible for variability in health outcomes. 

Furthermore, epidemiological studies on sewage workers exposed to bioaerosols are few and contradictory regarding the association between cases of illness and occupational exposure [46,47] because pathogens in sewage are widespread, especially in the case of adenovirus. However, serological studies in WWTPs have demonstrated that workers became rapidly immune to most of the pathogens after the first several months of employment, thus explaining the decrease in symptoms in the experienced workers [47]. Nevertheless, surveys performed in the past few years did not confirm occupational risk among sewage workers [48] because of the routine adoption of preventive measures which lower the real risk due to the reduction of the inhaled dose, thus determining the absence of significant differences in seropositivity toward pathogens among workers and general population. However, the presence of adenovirus in sewage pollution is widely documented, both in wastewaters and aerosol generated during sewage treatment [49,50,51,52]. In our work, adenovirus was chosen as the index pathogen owing to its abundance in sewage, persistence in the environment, and potential infectivity, which allowed us to make a conservative estimation of other pathogens levels in the environment.

## 5. Conclusions

The further design and proper application of QMRA models to bioaerosol exposure for occupational prevention will require a wide range of studies on methods, epidemiology, and monitoring to overcome limitations and to allow for more precise assessment. Future development of the QMRA model for occupational purposes should consider the difference in dose–response relationships based on gender or immunological status and also the deposition efficiency of aerosols in the respiratory tract in consideration of the inhalation route.
